# The ascension of nanosponges as a drug delivery carrier: preparation, characterization, and applications

**DOI:** 10.1007/s10856-022-06652-9

**Published:** 2022-03-04

**Authors:** Kartik Tiwari, Sankha Bhattacharya

**Affiliations:** Department of Pharmaceutics, School of Pharmacy & Technology Management, SVKM’S NMIMS Deemed-to-be University, Shirpur, Maharashtra 425405 India

## Abstract

Nanosponges are nanosized drug carriers with a three-dimensional structure created by crosslinking polymers. They have the advantage of being able to hold a wide range of drugs of various sizes. Nanosponges come in a variety of shapes and sizes. They are distinguished by the research method used, the type of polymer used, and the type of drug they may contain. Nanosponges are superior to other delivery systems because they can provide a controlled drug release pattern with targeted drug delivery. The period of action, as well as the drug’s residence time, may be regulated. Since it is made of biodegradable materials, it has a low toxicity and is safe to use. The efficiency of drug encapsulation is determined by the size of the drug molecule and the amount of void space available. Cancer, enzyme and biocatalyst carrier, oxygen delivery, solubility enhancement, enzyme immobilization, and poison absorbent are some of the applications for nanosponges. The method of preparation, characterization, factors affecting nanosponge development, drug loading and release mechanism, recent developments in this area, and patents filed in the area of nanosponges are all highlighted in this study.

**Figure:**
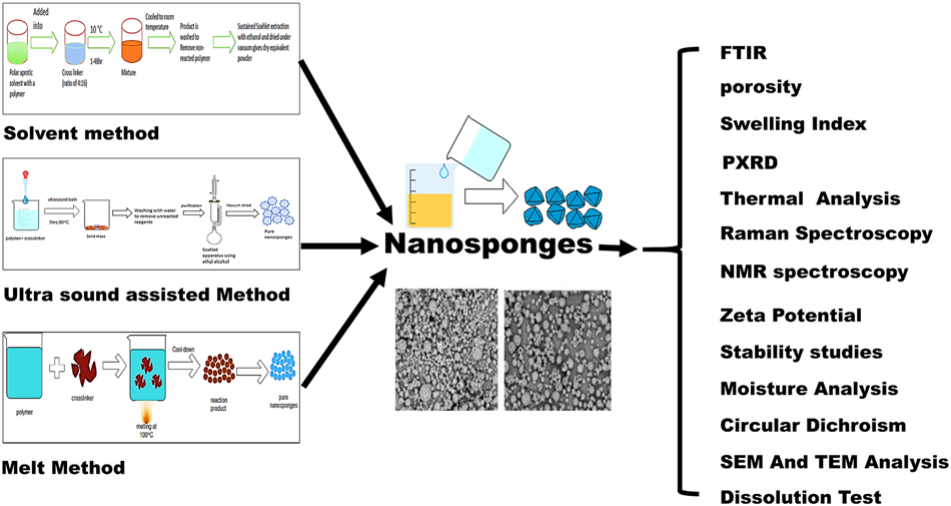
Graphical abstract

Graphical abstract

## Highlights

- The nanosponges are nanostructures that can carry small drug molecules.
- These can be administered by multiple routes and have a variety of applications.
- Variety of drugs with hydrophilic and hydrophobic characteristics can be administered for the treatment of many diseases.
- They are porous in nature, crosslinked by the use of multiple polymers.
- Their internal portion is porous in nature and has voids, which has the capacity to hold drug molecules.

## Introduction

This drug delivery system has a major accomplishment of providing a solution to the problems related to the release of the drug at the specific site at a determined rate [1]. Nanotechnology is possibly the most prominent engineering revolution since the industrial age [2]. Nanotechnology is defined as designing and alteration of materials at nanoscale levels to create products that shows advanced properties. A nanometer is a billionth of a meter. Nanomaterials are defined as physical substances that are having at least one proportion in 1–100 nm range [3]. Nanoparticles are fragments between 1 and 1000 nm in size with a neighboring interfacial layer [4]. These nanoparticles are accessible in various configurations like polymeric nanoparticles, hard- phospholipid nanoparticles, Nano emulsions, nanosponges (NSs), carbon nanotubes, micellar systems, dendrimers etc. The possible impression of nanotechnology considers the spatial and temporal scales, which is, the substance is engineered at the nanometer scale which can be controlled, and the individual constituents can be arranged to get the apparent substrate. Thus, it implies that the molecular synthesis and assembly of the nanoengineered substrates can be supervised providing specific and bulk chemical and physical properties. A broad range of substances like antineoplastic substances, proteins and peptides, volatile oil, DNA, and many more can be encapsulated in the colloidal structures termed as NSs [5]. The diameter of NSs is between 10–25 µm with a void space of 5–300 μm but the diameter of microsponges is less than 1 µm, hence NSs offer an advantage over the microsponges [6]. These NSs are sturdy up to a temperature of 300 °C, and in comparison, microsponges are firm but become fragile after crossing the temperature of 130 °C. The nature of NSs is lipophilic and introduced in water as a moving medium, which thus helps in masking unpleasant flavors and shows physical transition to liquid state from solid state [7]. NSs are an encapsulating type of nanomaterials that are composed of microscopic particles with little nanometer-wide cavities, hence providing a medium to incorporate and encapsulate a variety of drugs [8]. These materials are helpful in encapsulation of both, hydrophilic and lipophilic moiety, thus helping in upgrading the solubility of poorly water-solvable molecules [9]. According to the method of relating with active pharmaceutical ingredient, the nano sized particles can be categorized into capturing nano-sized particles, entangling nano-sized particles, and coupling nano-sized particles [10]. The treatment pattern of many diseases can be structured and rerouted because of the use of these miniature mesh-like structures. NSs provide us the advantage of delivering drug to the aimed site in a precise and foreseeable style [11]. They do not cause any mutations, irritations, allergic reactions or toxicity [12]. These are spongy spheres that have countless interconnected empty spaces called voids [13]. These voids help in entrapping a wide variety of drugs which for themselves are poorly soluble and encompass them in the matrix and thus improve their bioavailability. They have an inner lipophilic cavity with hydrophilic branching on the outside, which facilitates carrying both hydrophilic and lipophilic drug molecules [14]. These types of formulations can be used to mask displeasing flavor of the drug, which is possible by reacting cyclodextrin (CD) with an acceptable crosslinker, resulting in the formation of nano-sized materials having hyper-crosslinked CD called as NSs, and thus help to transform liquid substances into solid, thus giving the required result of masking of taste [15]. The use of crosslinkers in their structural morphology helps in targeted delivery of the drug. NSs are solid in nature and are found to be safe to be administered by other routes [16]. For administering parenteral formulations, aqueous solutions like saline and sterile water are used as solvents for incorporating NSs containing drug [17]. For administering drugs via topical route, topical hydrogel is used to incorporate the NSs. The topical NSs provide the advantage for greater patient compliance, reduced dosing and reduced side effects [18]. NSs when compared to nanoparticles, are not soluble in water or organic solvents, and are porous in nature, and are able to withstand temperatures up to 300 °C. They circulate around the body without sticking on any surface and thus initiate drug release. Multiple methods of preparation are available for NSs; (i) Solvent method, (ii) ultrasound-assisted synthesis, (iii) emulsion solvent diffusion method, and (iv) melting method. In the method called solvent method, polar aprotic dissolver like dimethylformamide is succeeded by further excessive addition of a crosslinker. On completion of the reaction, and cooling to an ambient temperature, it is transferred into large quantity of bi-distilled water, and then vacuum is used to filter and recover the final NSs [19]. In ultrasound-assisted synthesis method, polymer crosslinking is done by the use of ultrasonification. The ultrasonic waves are helpful in crosslinking of the polymer without the use of any dissolver. The mixture is then cooled and then washed with water to remove excess polymer [20]. The emulsion solvent diffusion method, the prime basis for the process is emulsification. Here, two phases that are not soluble in one another are used. Then dropwise, the internal phase is added, with constant stirring, into the external phase until internal phase disappears. Internal phase goes through evaporation process, and hence removed from the mixture, thus resulting in formation of NSs having minute pores [21]. In melting method, the melting of crosslinker and polymer occurs together. Then homogenization of all the ingredients is performed into fine state, heated at 100 °C for 5 h then cooled. The NSs are collected by repeated washing and then subjected to drug loading [22]. Some of the advantages seen with the use of NSs is the higher degree of entrapment efficiency (EE), which help in making NSs act as a pool for various pharmaceutical substances [23]. They also help in protecting molecules from degrading. The method of preparation is rather easy and regenerative in nature without much complications. They are also able to incorporate liquids into their 3D structure with ease [24]. Some of the disadvantages that are seen in such type of formulation are that only small-sized molecules can be entrapped especially having mass less than 500 Dalton (Da), so drug delivery of large-sized molecules is not feasible. Degree of crystallization affects drug loading [25]. There is also a possibility of dose dumping, which can be improved by use of polymer blends which will help in encapsulation of compounds with higher molecular mass [26]. 3D printing technology can help in NSs development which will thus help in providing NSs with modifications according to our needs [27]. The research which was studied projected an efficient tactic to the preparation of NS-based nano-sized carriers with multiple separate cavities with the ability to hold more than two Active pharmaceutical Ingredients accompanied by varying physicochemical characteristics. The three azole binders held in the NS assembly also strengthens the supramolecular joining ability to bioactive particles. In addition, mixtures have been tested for antiproliferative action [28].

## Types of nanosponge [29]

**Fig. 1:**
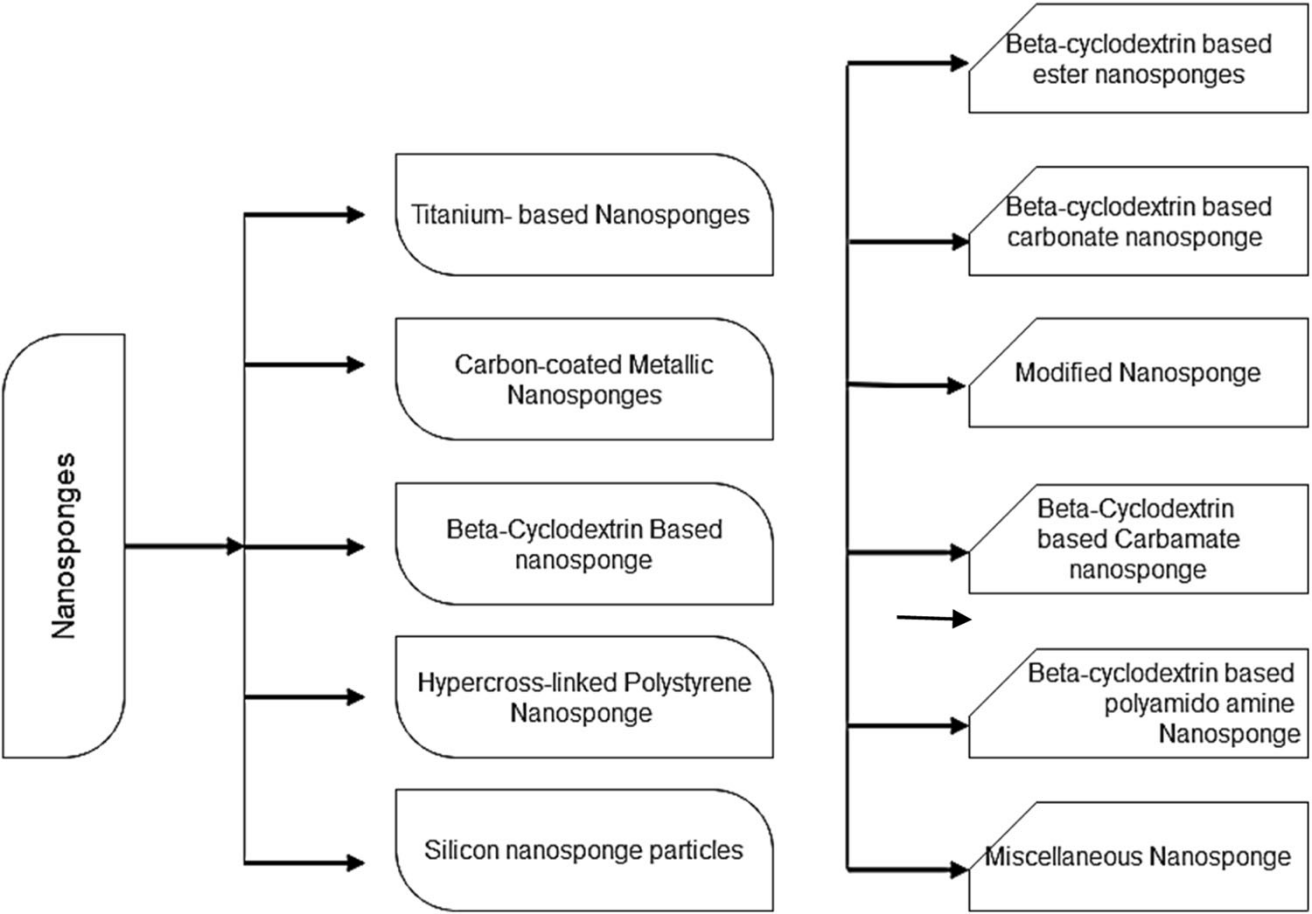
Types of nanosponges

There are many types of NS that are available and can be designed and formulated depending on the polymer added, its concentration, and the method of preparation used accordingly. The most common types of NS which are prepared and have been diversely used are beta CD-based NS. The formulation aspect for beta-CD NS is a relatively simple process and there are relatively multiple modifications that are possible (Fig. 1).

## Features of nanosponges

It is a type of encapsulating nanoparticle which is able to hold the drug molecule in the core [30]. The functional groups which are present in the crosslinker and concentration of the same affects the porosity of the NSs and gives flexible polarity [31]. The crosslinker helps in formation of cavities in the framework, thus providing the opportunity for modulation of drug release pattern [32]. NSs are nonlethal and steady up to elevated temperatures of approximately 300 °C [33]. Oral, parenteral, topical, and inhalational formulations are available as NSs preparations. Excipients like diluents, lubricants, anticaking agents, and the NSs are dispersed together in the form of matrix for the preparation of oral formulations [34]. In the process of preparation of topical formulation with the use of NSs, the NSs are mixed into topical hydrogel, and for formulations of parenteral use, they are mixed with sterile water, saline, Aqueous Solution [35]. The NSs have adherent property, where they can adhere to the surface and thus help in controlled drug release and in predictable manner. The drug release pattern is for 12 hours and hence, it gives the opportunity for incorporation of immiscible liquid which helps in improving the material processing and can be later converted into powder. They have good aqueous solubility which thus provides an opportunity for administering poorly water-soluble drugs [36]. They can carry equally, oil-loving and water-loving drugs. They have less side effects and are highly stable, and elegant. They have an improved formulation flexibility. They do not cause cancer, do not cause cancer, and do not cause allergies. They can be either in crystalline or para crystalline in nature. They give clear milky colored colloidal mixtures in water, and also have an added advantage of easy redevelopment by microwaves, solvent extraction, and thermal desorption method [37]. Exterior magnetic arena can be functional for precise release of the drug by accompanying of ferrite and magnetic agents during preparation [38].

## This type drug delivery system offers multiple advantages

The NSs help in covering the unpleasant taste of drug especially for the drugs which have to be administered orally, or by buccal route. The side effects of the various ingredients used during formulation can be reduced because they are getting encapsulated within the NSs [39]. They provide respectable formulation steadiness. They have self-sterilizing nature because the pore size is 0.25 micrometer and hence the bacteria cannot penetrate [40]. They are stable at pH of 1–11 range and temperatures up to 300 °C. Improved compatibility is seen with an extensive number of solvents and components. They help in modifying the rate of drug release from formulations [41]. Since the frequency of drug administration is decreased, there is an improved patient compliance. The scale-up process is easy and hence they can be easily commercialized. Both hydrophilic and lipophilic drug moieties can be encapsulated within the NSs [42]. Since there is use of crosslinkers during the formulation of NSs, they provide targeted drug delivery to the specific target. They can be easily redeveloped by methods such as washing, breaking with slightly inert gases, using nature-friendly liquids, slight increase in temperature, or by fluctuation in ionic or pH value [43]. The encapsulated drug is protected from the first pass metabolism because of the use of crosslinkers and other materials which are used in NSs formulation [44].

## Disadvantages of nanosponges as a drug delivery system

The NSs can incorporate small drug molecules. The drug loading capacity is affected by the degree of crosslinking since the crosslinking determines the void space available in the NSs which can be utilized in drug loading. There is a possibility of dose dumping due to early dissolution of crosslinker.

## Components used in preparation of NSs

Multiple compounds have provided positive results which can be used in the preparation of NSs and their use depends on the type of NS required and the with the level of crosslinking needed. The level of crosslinking is an important aspect in NSs, and depends on the concentration of crosslinkers used, since the drug release pattern and drug encapsulation depend on it. The compounds used in the method of preparation have been enlisted below (Table 1).

**Table 1:** Components used in preparation of nanosponges

Polymer	Copolymer	Crosslinker	Polar solvents
• HyperCrosslinked PolystyreneCyclodextrin (alkoxy carbonyl cyclodextrins)	• Poly(Valerolactone allyl Valerolactone)	• Carbonyl diimidazole	• Ethanol
	• Poly(Valerolactone allyl Valerolactone oxypanedione)	• Carboxylic acid dianhydrides	• Dimethylacetamide
• Methylβ-Cyclodextrin	• Ethyl cellulose	• Diarylcarbonates	• Dimethylformamide
• Hydroxy propyl β- cyclodextrin	• Polyvinyl alcohol	• Dichloromethane	
• Poly-Valerolactone		• Diisocyanates	
• Eudragit RS100		• Glutaraldehyde	
• Acrylic Polymer		• Pyromellitic anhydride	
		• 2,2bis(acrylamide)Acetic Acid	

## Method of preparation of nanosponges

### Solvent method

**Fig. 2:**
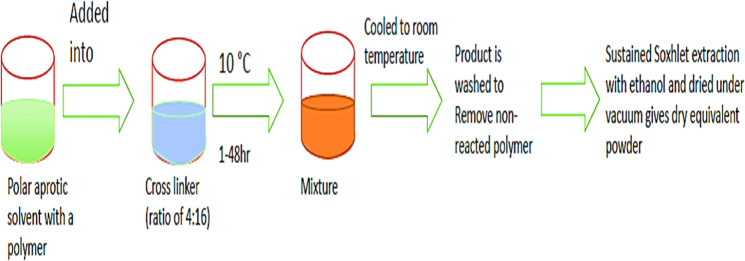
Solvent method

Suitable solvents, like dimethylformamide and dimethyl sulfoxide which are polar aprotic solvents, were used in the process. To this, polymer was added and properly blended. The crosslinker/polymer ratio of 8:2 is ideally used into which the above mixture was added. The mixture got from the above mixing, was then left to react for 48 hours and in a temperature range of 10 °C and up to solvent’s reflux temperature. On completion of the reaction, the solution was cooled until it reached the room temperature [45]. Excess amount of bi-distilled water was added to obtain the product from the above-cooled solution and the product was recovered under vacuum filtration (Fig. 2).

### Ultrasound-assisted method

**Fig. 3:**
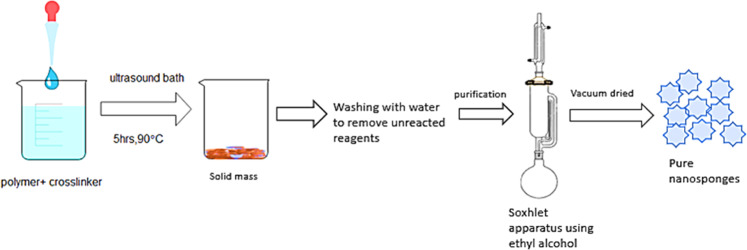
Ultrasound-assisted method

The ultrasound-assisted method of synthesis utilizes polymer ultrasonics junction. Crosslinking is got without using any solvent, and polymer crosslinking occurs due to ultrasonic waves. In a flask, polymer and crosslinker were combined at a reasonable molar ratio. During the ultra-sonication process, ultrasound bath was used to place the flask, at a temperature of 90 °C and for a time period of 5 h. The temperature of the collected mixture was reduced after sonication, and the product was split harshly and cleaned to extract unreacted polymer and reagents with an excess volume of water [46]. The washed solid was purified with ethyl alcohol by Soxhlet extraction. The filtered NSs acquired were vacuum dried and processed correctly until further loading of drugs (Fig. 3).

### Melt method

**Fig. 4:**
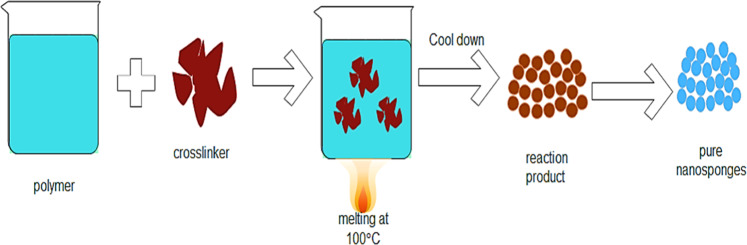
Melt method

The crosslinker and the polymer are melted together in the melting process. All the ingredients were finely homogenized. NSs were collected by washing the acquired product repeatedly with a suitable liquid. Cleaning the product, extracts the waste polymer and reagents which are unreacted and divides the product into the form of NSs [47]. Such blank NSs were further exposed to the encapsulating of narcotics (Fig. 4).

### Bubble electrospinning

A conventional and typical electrospinning configuration consists primarily of a syringe, syringe pump, as defined in many literatures, a high-voltage power, and a grounded collector. But one of the major limitations that limits their applications is the amount of output of nanofibers.

**Fig. 5:**
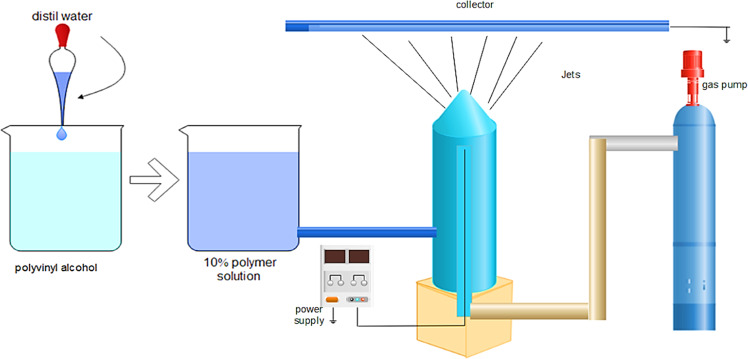
Bubble electrospinning

In bubble electrospinning, polyvinyl alcohol can also be used as polymer. By addition of distilled water into it, the solution of polymer (10%) was organized, which was then moved at 80–90 °C for 2 h to obtain a one-phase mixture. It was then left to achieve at room temperature with the polymer solution and then used to prepare nanoporous fibers [48] (Fig. 5).

### Synthesis by the use of microwave radiation

This is the simple technique of microwave irradiation synthesis of CD NSs that significantly decreases the reaction time. These NSs have higher crystallinity levels. Synthesis of NSs by microwave radiation showed a four-fold decrease in reaction time compared to traditional heating methods and also produced homogeneous particle size distribution with uniform crystallinity. Singireddy et al. [49] performed an experiment to ascertain beneficial effects of microwave-assisted heating in comparison to conventional heating during the synthesis of CD-based NSs. In the research, the outcomes suggested that NSs synthesized by microwave-assisted synthesis has doubled the drug holding capacity for the model drug. The results of high resolution-transmission electron microscopy (HR-TEM) displayed that the NSs obtained by microwave synthesis were highly crystalline, and showed increased degree of complexity along with narrow size distribution. The reaction time was greatly decreased for all reactions and the reaction products were improved under microwave-assisted heating conditions [49]. The benefit by means of synthesis using microwave irradiation is that it supplies straight energy to the targeted molecules and hence energy can be provided in precise form. The energy is not lost on heating the walls of the container or the liquid adjacent the reactant molecules and hence the full effect is seen in reaction progress towards completion. Zainuddin et al., [50] used microwave synthesizer to prepare β-CD in paracrystalline nature and used diphenyl carbonate (DPC) for crosslinking [51].

### Preparation of NSs from hypercrosslinked β-cyclodextrin

Arranged from β-CDs, their function as transporters for drug conveyance is carried out as nanosporous materials. Because of this 3-D structures, which could be a typically circular assembly about the extent of a protein with directs and openings in the inner portion, they are framed. For example, di-isocyanates, diaryl carbonates, carbonyl di-imidazoles, and so on, react to CD with a crosslinker [52]. The measurement of wipes is regulated by porosity, surface thickness of charge for the relation to different atoms. NSs are mixed depending on the crosslinker used in an impartial or acidic structure. They consist of solid particles and in the crystalline structure they have modified. Limit of NSs to demonstrate the tranquility and dissolvability of distinctive structures [53]. They are used to improve fluid dissolvability of drugs with insufficient water solvents [54].

### Emulsion solvent diffusion method

**Fig. 6:**
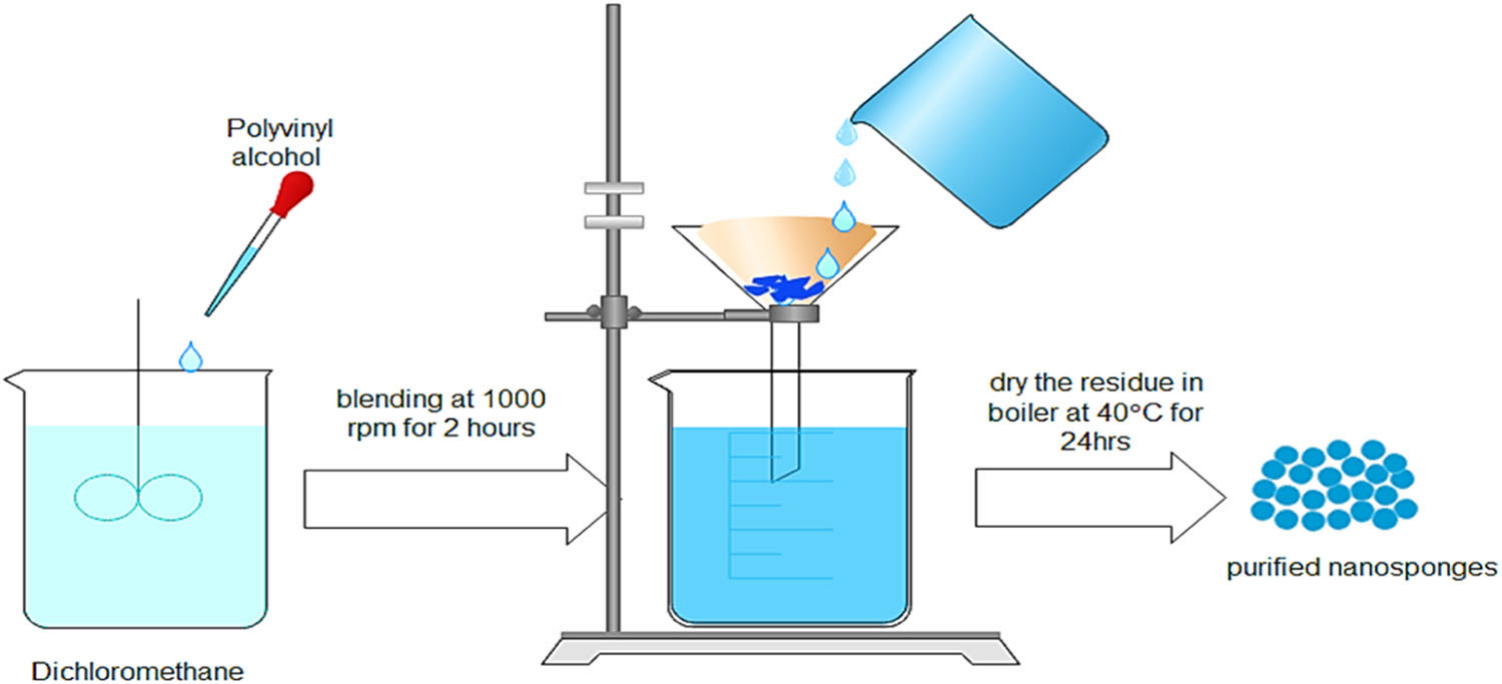
Emulsion solvent diffusion method

Two steps are used in this technique to vary the level of natural and aqueous (ethyl cellulose and polyvinyl liquor). In dichloromethane (20 ml) and an unmistakable measure of polyvinyl liquor added to 150 ml of fluid ceaseless process, the scattered stage with ethyl cellulose and moiety is dissolved. At this point, for 2 hours at 1000 rpm, the blend is thoroughly blended. The required NSs were collected by the filtration method and held for drying in an oven at 40 °C for 24 h. Dried NSs have been put away in desiccators and the evacuation of remaining solvents is assured [55] (Fig. 6).

Ilyas et al. [56] has prepared Naproxem sodium NSs using the solvent diffusion method and the diffusion rate was found to be close to 89% of some formulations and the drug loading efficiency to be close to 98%. They also studied the viscosity, particle size, zeta potential, and the stability studies. The Fourier Transform Infrared Spectroscopy (FTIR) results did not indicate any interaction between the drug and excipients. The results also specified high drug loading efficacy and exceptional release profile of drug [56].

### Quasi emulsion solvent method

**Fig. 7:**
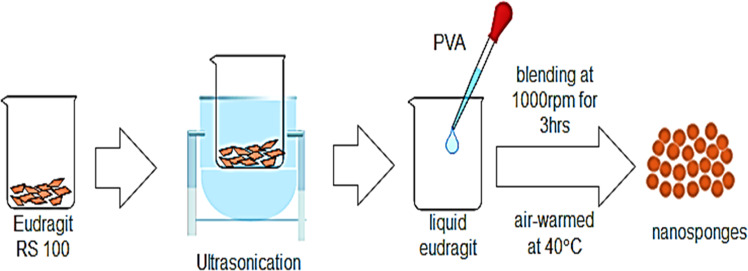
Quasi emulsion solvent method

The NSs were arranged in different sums using the polymer. Using Eudragit RS 100, the inner stage is prepared and added to a fair dissolvable stage. The drug used produced a response and broke down at 35 °C under ultra-sonication [57]. As an emulsifying operator, this internal process used in the outside phase containing polyvinyl alcohol goes around. At room temperature, the blend is blended at 1000–2000 rpm for 3 h and dried for 12 h in an air-warmed oven at 40 °C (Fig. 7).

## Ability to load drugs in nanosponges

The NSs are suspended in water and, to prevent aggregation, they are sonicated. Then centrifugation process is carried out on the dispersion, to get a solution of colloidal nature, which upon freeze drying, separates into supernatant and the NSs are dried. Then in the next step, drug, in excess amount, is dispersed and constantly stirred for a specified duration of time which allows the process of complexation to occur and thus aid in the formation of NSs in an aqueous suspension. After complexation, the un-complexed drug is separated by repeating the process of centrifugation [19]. Lastly, solvent evaporation method or freeze-drying method was employed to obtain the NSs [58].

## Mechanism of drug release from nanosponges

The NSs consist of multiple openings in their structures available in their core, which allow free passage of the drug molecule through and the liquid has achieved the state of saturation for the drug molecule. The end results are then enforced on the skin or taken internally, the moiety encapsulated achieves freedom to move into the vehicle and thus taken in by the skin [59], which contributes to the reduction of the drug concentration in the vehicle causing the state of unsaturation and thus disrupting the balance. This process follows until the complete medication have been absorbed by the body. The process studied above aids in assortment of vehicles suitable for the NS preparation. The solubility of the drug molecule increases in the liquid during the process of preparation which reduces the advantage of its gradual release, actually allowing the drug moiety to act like it had been added in its free form and not in its trapped form [24].

### Drugs loaded in nanosponges

There are multiple drugs that have been loaded in NSs and they have shown improved drug residence time in the body and hence less concentration of dosage form has to be administered. The drugs that have been administered in NS formulation are stated in the table below (Table 2).

**Table 2:** Drugs loaded in nanosponges

Class of drug	Drug
Antianxiety drugs	Lorazepam
Antiarrhythmic agents	Amiodarone hydrochloride
Antibiotics	Azithromycin, ciprofloxacin, erythromycin, ofloxacin, sulfamethoxazole, trimethoprim, Cephalexin
Anticoagulants	Warfarin
Anticonvulsants	Carbamezapine, clonazepam, felbamate, primidone
Antidiabetic and antihyperlipidemic drugs	Atorvastatin, fenofibrate, glibenclamide, Glipizide, nateglinide
Antiepileptic	Phenytoin
Antifungal	Econazole nitrate, Griseofulvin, Itraconazole, Ketoconazole, Lansoprazole, Voriconazole
Antihistamines	Terfenadine
Antihypertensives	Felodipine, nicardipine, nifedipine, telmisartan
Antineoplastic agents	Camptothecin, Docetaxel, Etoposide, Exemestane, Flutamide, Paclitaxel, Raloxifene, Tamoxifen
Antiretrovirals	Indinavir, Nelfinavir, Ritonavir, Saquinavir
Anthelmintics	Albendazole, Mebendazole, Praziquantel
Cardiac drugs	Carvedilol, Digoxin, Talinolol
Immunosuppressants	Cyclosporine, Sirolimus, Tacrolimus

## Recent advancements in nanosponges

There have been multiple improvements and advancements seen in the NSs drug delivery system over the past years. The list of drugs that have been loaded in them has also increased and so has their method of preparation. The EE and the type of polymers used as the components have also increased. The recent advancements seen in NSs have been mentioned in Table 3.

**Table 3:** Recent advancements in nanosponges

Name of researcher	Year of research	Title of project	Major finding	Conclusions
Monica R.P Rao, Rohini C. Bhingole	2015	Nanosponge-based pediatric-controlled release dry suspension of gabapentin for reconstitution	Important decrease in solubility; displayed the desired regulated profile at 12h; improved taste masking	Invivo tests show an improvement in bioavailability by 24.09% relative to pure drugs.
Trotta F, et al.	2016	Molecularly imprinted cyclodextrin nanosponges for the controlled delivery of L-DOPA: perspectives for the treatment of Parkinson’s disease	MIP-NSs show a slower, longer release profile; no degradation due to L-DOPA was observed for long-term storage at room temperature when integrated in MIP-NSs.	MIP-NSs are a promising option for L-DOPA storage and managed distribution.
Zainuddin R et al.	2017	Enhancement of oral bioavailability of Anti-HIV drug rilpivirine HCl through nanosponge formulation	Drug entrapment leads to improved solubility and twofold rise in drug breakdown following the Higuchi release model; enhanced oral bioavailability.	The strategy provides a convenient dosage range for patients with AIDs, minimizing the need for the consumption of medications only in a fed state.
Arvapally et al.	2017	Formulation and in vitro evaluation Of Glipizide nanosponges	The regression line from the Higuchi plot shows the drug release rate through the diffusion mode and further confirms the diffusion process.	The release kinetics of the optimized formulation was better suited to the Higuchi model and showed a zero-order drug release with the Fickian diffusion mechanism.
Momin M et al.	2018	Extended-release delivery of Erlotinib glutathione nanosponge for targeting lung cancer	Tumor volume decreased significantly; low doses of drugs are more effective over longer periods of time and are more specific to the target and thus minimize side effects.	Nanosponge can encapsulate high-efficiency anticancer drugs and exhibit extended release, have a higher antiproliferative effect than free drugs and thus display site-specific drug delivery and excellent cellular drug uptake.
Jin et al.	2018	Deoxyribozyme-nanosponges for improved photothermal therapy by overcoming thermoresistance	In vitro studies have shown that this nanosponge-ICG therapeutic platform can reduce the expression of HSP70 genes or proteins to normal levels during photothermal treatment and thus increase therapeutic efficacy.	Multivalent DNAzyme will effectively silence the HSP70 gene to heat MCF-7 cells, suppressing the heat shock response of cancer cells.
Wang et al.	2019	Nonviolent Self -Catabolic DNAzyme Nanosponges for Smart Anticancer Drug Delivery	DNAzyme nanosponges have been encoded with multivalent tandem aptamer that helps to deliver cancer cells efficiently; effective and accurate drug administration with synergistically enhanced therapeutic efficiency.	The present DNAzyme NS framework could be built to demonstrate outstanding applications in biomedicine and bioengineering.

## Factors influencing nanosponge formation

### Depending on property of crosslinker and polymer used

Crosslinkers assist in constructing a 3D structure of NSs. The degree of crosslinker used aids in determining the drug entrapment and also influences organ targeting [60]. The type of crosslinker used determines if the NS will be soluble in water or any other solvent [61]. Hydrophilic NSs are to be fabricated by the use of epichlorohydrin as a crosslinker. The advantage of using hydrophilic NSs in delivering of drug, is that it improves absorption of drug through the biological membranes and is a useful transporter for drugs to achieve immediate release formulations [62, 63]. The water-hating NSs are prepared using DPC, pyromellitic anhydride (PVA), diisocyanates, carbonyl imidazoles as crosslinkers. The hydrophobic NSs are used as a transporter of constant release drug delivery system for water-loving drugs counting peptides and proteins [64, 65].

### Medium used to show interaction and the property of drug

For selecting molecule which is apt for being incorporated in NSs have to have certain characteristics which make it suitable for encapsulation [66]. The drug molecule should have a molecular mass between 100 and 400 Da and below 5 condensed rings. The melting point should be below 250 °C and the solubility should be below 10 mg/ml in water [65]. The compounds with a high melting point have a lower stability constant value after entrapping in NSs and hence, uneven complexes are found between drug and NSs [67]. Also, the drugs with a high melting will be less entrapped and thus affect the drug loading capacity. The medium in which the drug loading is carried out also plays an important role in determining the rate of drug release, since it helps cause interaction between the cavities of the NSs and targeted compound [68]. If the medium is hydrophilic then the organic drug molecule gets entrapped into the hydrophobic cavities of NSs and if the medium is organic solvent, then it causes the release of organic molecules from the NSs [69]. The attraction between the guest molecule and host depends on physical and chemical attractions.

### The substitution degree for crosslinking

There is a direct relation between the degree of crosslinking and number of substitution where greater the number of substituents can provide higher degree of crosslinking thus yielding highly porous NSs having a mesh type networking in the internal structure [70]. A change in production process may lead to varying physicochemical properties in the material that formed, because of the functional group occupying different sites on the parent compound. The importance of degree of substitution of polymer depends on the ultimate value of the NSs which is dependent on the product processing and material purity [71].

### Complexation nature

The change in temperature affects the drug and the NS complexation. Constancy constant of the drug and the composite formed with the NS decreases with increase in temperature, which thus decreases the contact forces for example Van der Waal forces and the water-hating forces [72].

## Characterization

Multiple tests have been carried out on the NSs which test its strength, level of crosslinking and rate of drug delivery along with many other, which thus help in checking if the formulation is of suitable characteristics.

### Drug freight and snare proficiency

For determining drug loading, high concentration of drug is dissolved in to make a solution where the CD NSs are suspended. The dispersion is mixed and shaken at room temperature for a certain time period and then filtered to get the NS portion [73]. This portion is then freeze dried and the product got is used to calculate drug loading. For checking drug snare proficiency, the drug-loaded NSs are mixed in drug soluble liquid, then it is sonicated for disrupting the complex in NSs hence causing the drug to dissolve in solvent and the drug concentration in solvent is estimated by the analytical techniques such as UV–Vis spectroscopy and HPLC [74].

$$\% {Drug}\,{entrapment}\,{efficiency} = \frac{{{Drug}\left( {encapsulated} \right) \times 100}}{{{Drug}\left( {total} \right)}}$$

### Saturation state interaction

The equipment used for determining saturation state interaction is UV-Spectrophotometer. The increasing NSs concentration is added or mixed to fixed drug concentration and stored overnight. The formulation is scanned in UV range and the shift in absorbance maxima (λ _max_) in spectra is compared to that got by the pure drug [75].

### Phase dissolution studies

The phase solubility studies are used to study enclosure complexion which is demonstrated by Higuchi and Connors and thus detects the effects of NSs on dissolubility of drug [76]. The degree of encapsulation of the active pharmaceutical ingredient to the NSs can be considered by phase solubility diagram. Calculation can be done by excess drug moiety addition into suitable liquids until a soaked solution is prepared. In incremental concentration, the blank or empty NSs are added and then treated with saturated drug solution. More drug complexes with NSs and studied until balance is reached, as the NSs are added [77]. A graph is shown between the concentration of NSs and the concentration of drugs, and the graph is plotted which is described by the Higuchi and Connors classification. The constancy constant value portrays extent of NS and drug interaction, which if high, the rate of dissolution and solubility of poor water-soluble drugs increases [78]. Diagrams of phase solubility show the degree of complexity. Erlenmeyer flask was used for this test, into which the drug was poured and which already contained an aqueous solution of NSs in different concentrations and is then placed on a motorized mover and shaker at room temperature, for stirring [79]. The suspension of NS was then purified by centrifugation using a 3000 Da molecular sieve when a stable state was reached. The collected solution was analyzed by high-performance liquid chromatography (HPLC) to assess the drug concentration [80].

### Invitro release studies

The NSs are studied for their drug release pattern. The multi-compartment rotating cell is used where, aqueous NS-drug complexed dispersion was filled in the donor compartment and receptor compartment which is occupied with phosphate buffer for studies. Hydrophilic dialysis membrane acts as a separator between the two compartments. The receptor buffer is complete withdrawal of the receptor buffer was done periodically and filled with unsaturated buffer. The analytical method is used to calculate the amount of drug remaining and drug release [81].

### Porosity

This evaluation parameter gives the extent voids, of the NSs. For the study of porosity, helium pycnometer is used since the gas can flow between and through the channel mediums present in NSs. The material’s true volume is calculated by helium displacement [82].

$$\% {Porosity} = \frac{{{bulk}\,{volume} - {true}\,{volume} \times 100}}{{{bulk}\,{volume}}}$$

### Swell Index

The Brunauer–Emmett–Teller NS testing was performed using the N_2_ adsorption micrometric ASAP analyzer. Pre-heat treatment was given to the samples at 120 °C for 2 h before carrying out the study. At optimum temperature and after achieving equilibrium, a steady quantity of dehydrated NS sample (W_d_) was added to the bath. The exterior surface was then dehydrated and then measured using filter paper (W_h_). The procedure was performed three times, and an average W_h_ value was calculated [65].

$${{{\mathrm{Swelling}}}}\,{{{\mathrm{ratio}}}} = {{{W}}}_{{{\mathrm{h}}}}/{{{W}}}_{{{\mathrm{d}}}}$$

### Average diameter of nanosponges and their polydispersity

The average diameter is calculated by particle size analyzer instrument. Polydispersity is calculated by the principle of dynamic light scattering which is also known as photon correlation spectroscopy, helps in correlating the intensity variation of deflected light to particle proportions with auto-correlation function [83].

### Fourier Transform Infrared spectroscopy (FTIR)

The study of sample under FTIR shows structural elucidation especially the presence of the functional group in the structure. The range of detection for the same is from 4000 to 650 cm^−1^ for drugs, polymers, drug-polymer complex, blank NSs, drug-loaded NSs, and the possible interaction [84]. The FTIR results help in detection of hydrophilic and hydrophobic site in NSs. In hydrophobic drugs, if the functional group is not visible implies that the functional group has complexed with CD or the NS cavity. Bands of NSs slightly change after complex formation [85]. Infrared spectral studies provide details about hydrogen’s presence in different functional groups [86].

### Studies done by powder X-ray diffraction

Powder X-ray deflection helps in defining chemical breakdown and encapsulation. The pattern of diffraction differs when drug is complexed to a CD or NS and if there is alteration in crystallinity of drug [87]. The pattern of diffraction of a trial model is found as a scattering angle function. The resulting complex displays peak sharpening, and disappearance or appearance of peaks and their shifting [88]. When in solid state shows Inclusion Complexation, the detection is achievable. If the inclusion complex is in liquid state, there is no diffraction pattern found and hence prominent diffraction pattern helps differentiate between complexed NSs and newly developed substances [89]. When solid drug samples are studied, the diffractogram of complex that is expected is compared with that got from drug-polymer mixture. Comprehensive inclusion structure is ascertained by single-crystal X-ray analysis [90].

### Thermal analysis

The thermal analysis helps define the melting point (*T*_m_), temperature for crystallization (*T*_c_), crystallinity degree (*X*_c_), and thermal steadiness of NS-moiety complex along with pure drug [91]. Differential scanning calorimetry and differential thermal analysis studies checks widening, everchanging, arrival of new peaks or vanishing of peaks. Molecular mixture of polymer and drug complex is designated by shallow or disappearance of peaks. Weight variation tests are used to confirmed inclusion complex [92].

### Raman spectroscopy

In explaining the behavior of CD NSs, Raman spectroscopy is a useful technique when they move from dry to swollen state [93]. Raman peaks are delicate to the conformational variations of molecules and to intermolecular connections in terms of distance, strength, and wave number. It can also be used for the study of the structure of molecules. It also offers details about the state of water between the nanoporous structure and the dissolved solution. The dynamics of hydration can be studied by studying the vibration modes of decoupled OH and CH groups from the context of bulk water [94].

### Nuclear magnetic resonance (NMR) spectroscopy

NMR techniques like ^13^C,^1^H, 2D NMR, high-resolution Magic Angle spinning techniques help in understanding the structure of CD crosslinked polymers. The shift in chemical shift values (δ) indicates transfer of proton among species in reaction and hence ascertains structure of the NSs [95].

### Calculating the zeta potential of the nanosponges

The external charge of particle affects particle distribution and interaction and hence zeta potential is calculated. The parameters considered for calculating zeta potential are electrolyte concentration and pH. It can be measured in a particle size device by using additional electrodes. Samples of the NSs were thinned with 0.1 mol/L KCl for zeta potential determination and put in the electrophoretic cell, 15 V/cm electrical field was applied.

### Stability studies

In accelerated conditions and photodegradation experiments, the NSs have been subjected to stability studies. For 3 months, the formulation is tested annually. The changes in appearance, size, and physical characteristics, overall, the properties of the drug are investigated. The photodegradation analysis is carried out for 1 h under the UV lamp, stirring in the dark. At a distance of 10 cm from the lamp, the NSs are located. The sample is removed by HPLC and analyzed [96].

### To examine dampness of nanosponges

The non-water absorbent role of NSs is established by dynamic vapor absorption studies and concerned preservation of crystalline assembly while adsorption and elution of dampness [97].

### Molecular modeling studies

During the study of NSs under the molecular modeling studies, very dense assembly in dry state is perceived by molecular dynamics simulations of a NS prototype. The simulations display the swelling of NSs [98].

### Circular dichroism

The use of circular dichroism spectroscopy is to assess the presence of CD inclusion compounds especially in aqueous solutions. The circular dichroic absorption requires chirality and electron optical absorption. Indeed, it was noticed that placing an achiral guest molecule into an asymmetric CD cavity produced extrinsic Cotton effects in the guest molecule’s absorption band, as calculated by circular dichroism. An outer surface association between guest molecules and CDs, on the other hand, only modifies other spectral properties without triggering the Cotton effect [99].

### Scanning electron microscopy (SEM) and transmission electron microscopy (TEM)

For the analysis of topography of drug surface, NSs, and the product (drug/NS complex), SEM and TEM can be used. The alteration between the state of crystallization shown by initial materials and the resultants were observed under the electron microscope which suggests that inclusion complexes are formed. The NS surface morphology was determined using the JEOL JSM-5610LV scanning electron microscope for 30 kV transmissions. JEOL JFC-1600 auto fine coater helps with covering of the product with gold-palladium alloy, and hence the sample was made. In another analysis, TEM JEOL 1400 was used at transmissions of 60 kV where approximately 10 μL of NS sample was diluted with Milli-Q water to 100 μL. In order to visualize the sample, 5 μL of the watery mixture, the sample was held on a network which was then held on glass plate for microscope and then microscopically observed [100].

### Resilience tests

It is possible to adjust the toughness (viscoelastic properties) of NSs to create softer or firm beadlets according to the final product requirements. Increase in crosslinking gradually slows the release rate. Therefore, by consideration of the release pattern, as a function of crosslinking with time, the resilience of sponges can be studied and optimized as needed [101].

### Dissolution test

The dissolution profile of NSs can be analyzed using the USP XXIII dissolution apparatus with the aid of modified basket consisting of 5 m stainless steel wire, with a rotation speed of 150 rpm. The selected dissolution medium ensures sink conditions is maintained when studying the solubility of active compounds. The final samples are analyzed by available analytical techniques [102].

## Applications of nanosponges

### Nanosponges for drug delivery

NSs can favorably bear drugs which cannot dissolve in water because of their composition containing voids. The mixing rate, dissolubility and constancy of moiety, the masking of undesirable tastes and the conversion of liquid substances to solids can be improved by using these complexes. β-NSs based on CD are stated to transport the drug three to five times more effectively to the target site than direct injection. In nature, the NSs are hard and hold potential to be designed as pharmaceutical form of inhalation, oral, parenteral and topical. Complexes can possibly be distributed in a mixture of lubricants, thinners, excipients and anticoagulants appropriate formulation tablets, capsules aimed at oral administration [103]. The transportation of the complex can be efficiently done in saline, sterile water or other liquids for parenteral drug delivery. Drug can be efficiently encapsulated into a hydrogel for topical administration.

### Nanosponge as a transporter of biocatalysts, enzymes, proteins, antibodies, and vaccines

Operational drawbacks are associated with many manufacturing processes that require chemical transformation. Reactions with no specific end point, showing yield of less quantity, and the constant requirement to work at high conditions in the downstream process requires the consumption of great quantities of energy and huge quantities of water for cooling. By utilizing enzymes as biocatalysts, all these disadvantages can be removed or greatly decreased. Under mild reaction circumstances, these enzymes work, have high speed for reaction, and are high specificity. They have a useful environment impact, since they reduce the utilization of energy and decrease pollutant output. The enzyme’s catalytic action rests on principally, proper orientation of the active site. In the biomedical and therapeutic sectors, there is use proteins, peptides, enzymes and their close imitative. Catalytic adjuvant can be utilized for treating tumor or type I mucopolysaccharidosis, while gene therapy utilizes DNA and oligonucleotides. There is presence of multiple issues and drawbacks about the administration of bulky molecules. Due to certain points, like bulky molecular dimensions, water-loving nature, excitation ratio, large value of topographic charge, synthetic and enzymatic variability and small entering capability via mucous membranes, and multiple drugs made of protein are badly taken in via biological membranes. Protein molecules can be easily removed from the blood following intravenous administration, join to plasma proteins, and are responsive to enzymes that can cause breakdown. Bioavailability is a major concern for oral drug administration [104]. For medicinal usage, different methods exist, such as swelling the dosage or using captivation promotors, which may cause concerns with toxicity. A variety of schemes, such as nanoparticles and microparticles, liposomes and hydrogels, have been developed to hold enzymes and proteins. Carriers may guard proteins from degradation, alter their pharmacokinetics and enhance their in vivo steadiness in a specific system. CD-based NS have now been found to be especially appropriate carriers for adsorbing antibodies, enzymes, proteins and macromolecules. Specifically, when enzymes are used, their function, performance and operation can be retained, and the pH and temperature spectrum of activity can be expanded and continuous flow processes can be carried out. In addition, proteins and other macromolecules can be transferred to CD NS by adsorbing or encapsulating them. Stability is not shown by bovine serum albumin (BSA) in solution of polypeptide. The storage is in lyophilized condition. However, proteins may be unpredictably denatured by lyophilization and adopt configuration that is substantially different from native proteins structured. The key drawback in the formulation and production of proteins is to retain its native structure during processing and long-term storage. Applicability of NS-based proteins like BSA are complexed in swell-capable CD-based poly (amidoamine) NS, which improves stability of protein. NS have also been used for protein encapsulation, enzyme immobilization and subsequently stabilization and regulated delivery. Oxidoreductase, transferase, hydrolase, lyase, isomerase and ligase groups are the enzymes which have been studied. Complexation of model BSA has been done with NS, and studies have indicated extended release of albumin. Along with that, the encapsulation of albumin in the NS preserved the protein and stabilized it during storage [105].

### Gaseous encapsulation

CD NSs based carbonates has been employed to form enclosure complexes with three different gases, i.e., 1-methylcyclopropene, oxygen and carbon dioxide. Carbon dioxide and oxygen complexion can be beneficial in many biomedical applications, wherein, NSs filled with oxygen can effectively deliver to the tissues that become hypoxic [53]. Due to their highly porous nature, the NSs were also explored as a gas carrier, with oxygen release in a controlled way. In the future, they are capable of being a valuable tool for the absorption of such gases. Alpha, β-, or gamma-CD, have been found capable to capture O_2_ efficiently for a long duration. NSs of such type are capable of releasing O_2_, with or without ultrasound. However, the results of an in vitro release study have shown that ultrasound improves liberation and entry of O_2_ into the cell. The results of the study suggested that NSs can be effectively used as an O_2_ reservoir and carrier for its topical delivery. In the results of another research, alpha-CD-based NSs were evaluated for their in vitro O_2_ release and oximeter was used to calculate. Cell mortality reduced when formulations were given prior to hypoxia attacks and observations were made in contrast with oxygen-free samples. Extended and continuous release of O_2_ was observed from alpha-CD NSs with potential of controlled oxygenation [106].

### Cancer treatment

Sometimes, in cancer patients, medications injected by physicians are made ineffective. This occurs mostly for two reasons: either they are unable penetration in the tumor site, or the immune system assaults and dismembers them. This obstacle has now been overcome to a certain degree by the use of NS. Experts indicated that fixing NS drugs guaranteed that the substance reach their destination in significant quantities. In Paclitaxel, the active ingredient is Taxol which is useful in the anticancer treatment, and is an effective medication which has been developed as a NS formulation [107]. In animal studies, the researchers reported of two distinct tumor types which showed response to single injections of NS formulations: one is the slow-growing human breast cancer and other is fast-acting mouse glioma. In both cases, the delivery of NSs has been shown to improve cancer cell death and slow tumor growth relative to other chemotherapy methods. Camptothecin (CAM), has a lactone ring which shows low aqueous solubility and thus low therapeutic benefits. Lactone ring opens up physiological pH and converts into inactive carboxylate which causes concerns of unpredictability and severe after effects. CD-based NSs are a crosslinked derivative of CDs. The introduction of CAM into the NS has led to a improved release profile in active form showing a longer release profile, which avoids the breakdown of the lactone form and thus shows stability improvement [107]. An extract derived from the dried roots of rhizome curcuma is curcumin (CUM), which has successfully shown potential to be used in treatment for tumors. NSs formulated with CUM provide resourceful dispersal of complexed CUM, improved solubilization performance and stability because of reduced possibility of hydrolytic degradation and biotransformation over wide time duration [108].

### Delivery system for oxygen

A system to deliver oxygen was developed by the use of CD and formulated as NSs. NSs consisting of alpha, β and γ-CD are mixed in aqua, then soaked with oxygen, and characterized in vitro for this purpose [109]. Oxygen infusion through a silicone film is achievable by the use of β-CD NS/hydrogel combination device. Over time, it has been seen in research that NS hold the capacity to release oxygen in a controlled pattern. Oxygen encapsulated NSs can stick to oxygen deficient cell mass that are present in different diseases with oxygen.

### Rare blood cancer marker harvesting

NSs with the interior adorned with various types of bait-molecules, have shown to be useful to selectively deceive unique blood protein families and shield them from blood enzyme degradation [110].

### In the elimination of organic matter to provide ultrapure water for the regeneration of power

For the industries that require very clean water, like the pharmaceutical industries, the presence of natural contaminants in water is an alarming concern. The water-insoluble CD polymers play an important role and are efficacious in discarding waste from water which is collected at a particular power plant. The aptitude to extract liquified organic carbon from raw water by as much as 84% has also been shown by the CD polymers, though total organic carbon removal was relatively poor [111].

### Enhancement of solubility

NSs have also been used to improvise the dissolvability and dissolution rate of drugs with low solubility and thus provide a regulated release profile. Molecular magnitudes and contour, however, are essential constraints affecting the complexity of inclusion in-bound NSs and therefore may not be uniformly relevant to all moieties. Cefpodoxime proxetil NSs have been prepared to increase the dissolution rate of the same [112].

### System of topical supply of drugs

Some of the formulations for external use can be local anesthetics, antifungals, and antibiotics which show convenience to be formulated as NSs [29]. Hence, NSs can be arranged using a variety of methods, such as emulsion solvent diffusion method, etc. Econazole nitrate NSs, which are discreetly smooth nano-sized elements with pierced orange peel, have been prepared as visualized by SEM in the literature [113].

### To boost EVA combustion properties, by use of novel flame retardants containing cyclodextrin nanosponges and phosphorus compounds

In this context, the availability of acidic source, loss of water is seen in CD NSs, producing water vapor and char, thus protecting the copolymer against combustion [114].

### Antiviral application

Drugs that are currently employed in nano-supply device are zidovudine, saquinavir, interferon-5-007, acyclovir (Eudragit based) [110].

### In enzyme immobilization

Recent studies have confirmed the highly hydrolytic efficiency shown by Pseudomonas fluorescence lipase when adsorbed to a new type of CD-dependent NS [115].

### Protective agent against photodegradation

NSs are prepared by encapsulating gamma-oryzanol with strong protection from photodegradation [116].

### Modulation of drug release

The key downside to most traditional, commercially available drug delivery systems is constant administration. However, a drug that is loaded into a NS is stored and released slowly over time. Hydrophobic CD NSs are used as a continuous release carrier for water-soluble drugs, including peptide and protein drugs [73].

### As absorbent in treating poison in blood

NSs are used to extract harmful compounds that are detrimental to the blood by removing the toxin. NSs can absorb toxins when given by injection and are better than antidotes. NS appears like a red blood cell in a bloodstream that tricks toxins into targeting and consuming them. NSs can absorb toxin molecules depending on the toxin [117].

### Role of nanosponges for the treatment of fungal infections

One of the most feared diseases in the world is fungal infection. For the treatment of these cutaneous infections, topical treatment is an important option because of its multiple benefits, such as directing drugs to a specific disease site and reducing its adverse effects. An effective antifungal or fungicide that is topically used to treat various skin diseases such as vaginal thrush, tinea pityriasis Versicolor, ringworm, jock itch, and athlete’s foot is econazole nitrate which is available in the form of cream, ointments, lotions, and solutions. Econazole nitrate is only effective when mixed with a high concentration of active substances and applied to the skin. To this end, the use of emulsion solvent system econazole nitrate NSs was manufactured and then injected into a hydrogel to release the drug in a continuous manner as a topical application.

### Use of nanosponges as a diagnostic tool

β-CD is widely used for the manufacture of different diagnostic materials. The properties provided by CD NSs such as high biocompatibility, prolonged circulation of the blood, uniform size distribution for permeability and easy access to the target make them excellent for use as a diagnostic agent [63].

### Use of nanosponges in cosmetics

NSs has a range of uses in the cosmetics industry. NSs provide good protection for cosmetic ingredients that are prone to photodegradation. It can catch up and prolong the release of Volatile oils, guy. The bad smell of the body created by sweating can also be absorbed. It can release volatile ingredients slowly, thus providing a long-lasting fresh feel in oral cosmetics. It can also be used in items such as rouge or lipsticks to have a long-lasting effect.

## Patents

Recently, there have been new patents that have been filed and approved in the field of NSs where they have been employed to improve the method to preparation which thus makes the process efficient. The patents have been filed with their use as toxin absorbing agents, growth preservation, enzyme release and biocatalyst studies. They have also shown promising results as antitumoral agents. The new patents are also granted from the authority which helps to shift the demand towards NSs as a novel drug delivery system and have been described below (Table 4).

**Table 4:** Recent patents

Sr. no.	Patent/App. No.	Applicant	Title
1	W02006002814A1	Francesco Trotta, Wander Tumiatti, Orfeo Zerbinati, Carlo Roggero, Roberto Vallero	Ultrasound-assisted synthesis of cyclodextrin-based nanosponges
2.	W02009149883A1	Gianfranco Gilardi, Francesco Trotta, Roberto Cavalli, Paolo Ferruti, Elisabetta Ranucci, Giovanna Di Nardo, Carlo Mario Roggero, Vander Tumiatti	Cyclodextrin nanosponges as a carrier for biocatalysts, and in the delivery and release of enzymes, proteins, vaccines and antibodies.
3.	ITMI20071321A1	Giovanni Nicolao Berta, Roberta Cavalli, Barbara Mognetti, Carlo Maria Roggero, Francesco Trotta, Vander Tumiatti	Nanosponges based on cyclodextrins as a vehicle for anticancer drugs
4.	W02012147069A1	Universita DegliStudi Di Torino, Sea Marconi, Technologies Di	Method of preparing dextrin nanosponges
5.	CA2692493A1	Sea Marconi, Technologies Di Vander Tumiatti, S.A.S, Francessco Trotta, Vander Tumiatti, Roberta Cavalli, Carlo Mario Roggero, Barbar Mognetti, Giovanni, Nicolao Berta	Cyclodextrin-based nanosponges as a vehicle for antitumoral drugs
6.	ITTO20110873A1	Vecchi Marco DeCarlo Stefano DiShubhen KapilaCarlo Maria RoggeroValentina ScariotMichela Tumiatti	Use of nanosponge functionalized for growth, preservation, protection and disinfection of plant organisms
7.	US9574136B2	Kun Lian	Nanoparticles, nanosponges, methods of synthesis, and methods of use
8.	US20170152439A1	Kun Lian	Nanoparticles, nanosponges, methods of synthesis, and methods of use
9.	US8828485B2	Kun Lian, Qinglin Wu	Carbon-encased metal nanoparticles and sponges as wood/plant preservatives or strengthening fillers
10.	WO2007095454A2	Kun Lian, Qinglin Wu	Carbon-encased metal nanoparticles and sponges, methods of synthesis, and methods of use
11.	WO2009138998A3	Eswaramoorthy Muthusamy, Saikrishana Katla	A template free and polymer free metal nanosponge and a process thereof
12.	WO2012147069A1	Francesco Trotta, Pravin SHENDE, Miriam BIASIZZO	Method for preparing dextrin nanosponges

## Toxicological studies

Toxicological assessment is a significant step in assessing the drug and excipient safety; along with assisting in choice of appropriate dosage form for humans and animals use. The important lead is that the destiny of the parent CDs in the gastrointestinal tract is decided by hydrolysis resistance and enzymatic degradation. There is resistance seen of Beta CDs to gastric fluid and amylases from pancreatic fluids and thus reach colon for complete hydrolysis. An evident proof was a study including healthy volunteers and ileostomists, that beta CDs are entirely digested in the colonic microflora, but are almost substandard breakdown in the human small intestine. In the human colon, the enzymatic degradation of beta CDs may be carried out by either glucose or maltodextrins. After oral administration to rats, the LD50 of beta CD is calculated to be 18.8 g/kg [118]. Previous in vitro experiments have been performed to determine the safety of NS where they did not display hemolytic activity or cytotoxicity tested on various cell lines. Additional evaluation was carried out for the invitro stability of NSs against chemical degradation. It was observed that there was initial deterioration of the NS structure within 2 h when the NS were subjected to Acidic conditions (0.1 N HCl) due to which there was restricted release of cyclodextrin units and when the NS were subjected to basic environment (NaOH 0.1 N), there were no visible effects on the stability of the NSs. Conversely, using PMDA (pyromellitic dianhydride) as a crosslinker for preparing NS, tends to lessen the structural integrity in simple solutions, even though it preserves its structure for 24 h. The starting dose of 300 mg/kg when tested for the LD50 test, and the results showed lowest toxicity following oral administration. Control and treatment groups showed no cases of mortality, at all available level doses during the study period. A dose of 2000 mg/kg body weight of NS formulation was selected as the dose with low toxicity and was decided according to the results of the recent study, and the dose also did not show any unobserved adverse effects [119]. Liver toxicity is associated with major changes in enzymes, such as ALP, AST and ALT. Liver is the site of cholesterol and protein synthesis, so a change in the level of cholesterol also suggests the toxicity of the liver. Glucose regulation is done by liver and it produces free glucose from hepatic glycogen stores, which is main store of cholesterol production and removal. There have been no alterations for 28 days after administration of compounds, which suggest no significant damage to the liver. The change in biochemical parameters like urea and creatinine suggest if any kidney damage has occurred. Thus after 28 days of administration of NS formulation, the above parameters remained normal. As a result, renal toxicity and hepatotoxicity was not induced from the administration of NS formulations.

## Future trends

The advent of NSs, which have been shrunk down to nanoscale, has ushered in a revolution in medical science. In the field of medical science, a revolution has started as technology has been used to get to the nano-scale. When a targeted and managed drug release mechanism is used, drug toxicity is reduced because improved therapeutic results can be achieved. The role of NSs in the field of therapeutics and nanotechnology growth is critical. In the future, NS could be used as a standard water purifier. The main problem is lowering production costs, which will necessitate the exploration of new polymers and crosslinkers as well as the establishment of new production methods. Because of their unique nature, they play an important role in downstream production, necessitating extensive research. The influence of particle size, synthesis, crystallinity, porosity, and degree of crosslinking on drug release holds great promise. Up to this point, the most commonly recorded methods of preparation have been ultrasound-assisted synthesis and the traditional approach, but new methods such as bubble electrospinning and solvent evaporation are also being updated and developed. The new trend is to increase yields, cost-effective production, and reproducibility, both of which will help with mass production in a short period of time. While current NS preparation methods are simple, a major flaw in the chemical process is the presence of residual liquids or reaction leftovers in the final product, which can be a very likely cause of toxic effects [115].

## Conclusion

The NSs provide a flexible drug transportation medium as well as a controlled intermediate for drug delivery to the specified area. They can encapsulate a wide range of medications, allowing for oral and topical drug delivery. There is also the benefit of transporting and encapsulating both lipophilic and hydrophilic drugs, as well as a wide range of applications in areas such as solubility enhancement, entrapment of gases such as oxygen, cosmetics, diagnostics, and poisoning adsorbents. Multiple assessment tests are performed to determine the structural and chemical integrity of the product. They will be useful in a variety of fields in the future, and their scope of application will expand as research in this field expands.

## Data Availability

Data will be available on request.

## Abbreviations

**AIDS**: Acquired Immunodefficiency Syndrome
**BSA**: bovine serum albumin
**CAM**: camptothecin
**CDI**: carbonyl imidazoles
**CUR**: curcumin
**DPC**: diphenyl carbonate
**EE**: entrapment efficiency
**FTIR**: Fourier Transform Infrared Spectroscopy
**HPLC**: high performance liquid chromatography
**HR-TEM**: high resolution transmission electron microscopy
**HSP70**: heat shock proteins 70
**L-DOPA**: levodopa
**MCF-7**: Michigan Cancer Foundation-7
**MIP**: NSs: molecular imprinted nanosponges
**NSs**: nanosponges
**PMDA**: pyromellitic dianhydride
**PVA**: pyromellitic anhydride
**SEM**: scanning electron microscopy
